# Clinical and Virological Factors Associated with Viremia in Pandemic Influenza A/H1N1/2009 Virus Infection

**DOI:** 10.1371/journal.pone.0022534

**Published:** 2011-09-27

**Authors:** Herman Tse, Kelvin K. W. To, Xi Wen, Honglin Chen, Kwok-Hung Chan, Hoi-Wah Tsoi, Iris W. S. Li, Kwok-Yung Yuen

**Affiliations:** 1 State Key Laboratory for Emerging Infectious Diseases, Carol Yu Centre for Infection, The University of Hong Kong, Hong Kong, China; 2 Department of Microbiology, The University of Hong Kong, Hong Kong, China; 3 Department of Microbiology, Queen Mary Hospital, Hong Kong, China; Blood Systems Research Institute, United States of America

## Abstract

**Background:**

Positive detection of viral RNA in blood and other non-respiratory specimens occurs in severe human influenza A/H5N1 viral infection but is not known to occur commonly in seasonal human influenza infection. Recently, viral RNA was detected in the blood of patients suffering from severe pandemic influenza A/H1N1/2009 viral infection, although the significance of viremia had not been previously studied. Our study aims to explore the clinical and virological factors associated with pandemic influenza A/H1N1/2009 viremia and to determine its clinical significance.

**Methodology/Principal Findings:**

Clinical data of patients admitted to hospitals in Hong Kong between May 2009 and April 2010 and tested positive for pandemic influenza A/H1N1/2009 was collected. Viral RNA was detected by reverse-transcription polymerase chain reactions (RT-PCR) targeting the matrix (M) and HA genes of pandemic influenza A/H1N1/2009 virus from the following specimens: nasopharyngeal aspirate (NPA), endotracheal aspirate (ETA), blood, stool and rectal swab. Stool and/ or rectal swab was obtained only if the patient complained of any gastrointestinal symptoms. A total of 139 patients were included in the study, with viral RNA being detected in the blood of 14 patients by RT-PCR. The occurrence of viremia was strongly associated with a severe clinical presentation and a higher mortality rate, although the latter association was not statistically significant. D222G/N quasispecies were observed in 90% of the blood samples.

**Conclusion:**

Presence of pandemic influenza A/H1N1/2009 viremia is an indicator of disease severity and strongly associated with D222G/N mutation in the viral hemagglutinin protein.

## Introduction

The pandemic caused by the influenza A/H1N1/2009 virus has caused significant morbidity and mortality in certain demographic groups, like the extremes of age, pregnant women and obese patients [1], [2]. The clinical, virological and immunological parameters associated with clinical severity have been explored in recent studies [3]. Among them, delayed clearance of viral load, immuno-dysregulation as assessed by chemokine activation and lower IgG2 levels were noted to be associated with greater clinical severity and complications [4], [5]. Coincidentally, increasing evidence linked a mutation, D222G (D225G in H3 numbering), in the hemagglutinin (HA) of the pandemic influenza A (H1N1) 2009 virus to severe disease [6], [7], [8], [9]. In a recent study, we showed a significantly greater population of quasispecies with D222G substitution in the lower respiratory tract as compared to that of the upper respiratory tract [10].

Although the influenza virus is primarily a respiratory pathogen, clinical manifestations in severe infections are not necessarily restricted to the respiratory tract and more closely resemble those in a systemic infection. In fact, positive viral RNA detection outside the respiratory tract was often observed in severe pandemic influenza A/H1N1/2009 virus infections [4], [11]. The condition is reminiscent of viremic cases in severe human infections caused by the avian H5N1 influenza virus [12], [13]. For seasonal influenza, naturally occurring human cases of viremia have also been reported [14], [15], [16], [17], [18], [19], but most of the results date back to more than three decades ago and viremia in seasonal influenza has been largely overlooked in recent years. Hence, our present understanding of viremia in human influenza infection remains limited.

We investigated the clinical significance of viremia in influenza A(H1N1) 2009 virus infections, and any possible relationship between viral genotypes and dissemination outside the respiratory tract. Sequence analysis was performed on viral RNA detected from different samples and results showed that variants carrying HA D222G/N substitutions were significantly associated with viremia. The presence of viremia was associated with a more severe clinical presentation and possibly higher mortality.

## Methods

### Ethics statement

The study was approved by the Institutional Review Board of the Hospital Authority in Hong Kong. The named ethics committee specifically waived the need for consent as this was a retrospective analysis.

### Patients and clinical data

Adult patients admitted to hospitals in Hong Kong between May 2009 and April 2010 inclusive and tested positive for pandemic influenza A/H1N1/2009 virus in their respiratory specimens were included in the study.

Severe cases were defined by the patient experiencing oxygen saturation of <90% while breathing room air and requiring respiratory support or admission to an intensive care unit. Patients with mild disease were randomly selected from those who did not experience oxygen desaturation and were not admitted to an intensive care unit. Patients without archive blood samples were excluded. Clinical data were retrieved via a retrospective review of medical records.

### Molecular detection and characterization of influenza virus

Viral RNA was detected by reverse-transcription polymerase chain reactions (RT-PCR) targeting the matrix (M) and HA genes of pandemic influenza A/H1N1/2009 virus from the following specimens: nasopharyngeal aspirate (NPA), endotracheal aspirate (ETA), blood, stool and rectal swab. Stool and/ or rectal swab was obtained only if the patient complained of any gastrointestinal symptoms, including abdominal pain, nausea, vomiting and diarrhea. RT-PCR targeting the influenza A M gene, pandemic influenza A H1, and seasonal influenza A virus H1 and H3 gene, were performed as previously described [20], [21], [22]. Briefly, total nucleic acid extraction was performed using NucliSens easyMAG instrument (bioMerieux; Durham, NC). The primer sequences were as follows: M gene forward primer: 5′-CTTCTAACCGAGGTCGAAACG-3′; M gene reverse primer: 5′-GGCATTTTGGACAAAKCGTCTA-3′; Real-time one-step RT-PCR assays were used for the detection of pandemic influenza A/H1N1/2009 virus, seasonal influenza virus H1 and H3 using Invitrogen SuperScript III Platinum One-Step Quantitative Kit in a 7500 Sequence Detection System (Applied Biosystem, Foster City, CA, USA) with forward primer 5′- CCAAAGCTCAGCAAATCCTACAT-3′, reverse primer 5′-GATGGTGAATGCCCCATAGC-3′, and probe Fam-TGATAAAGGGAAAGAAGTCCT-MGB. seasonal influenza virus H1gene forward primer: 5′-AACTACTACTGGACTCTRCTKGAA-3′; seasonal influenza virus H1 gene reverse primer: 5′- CCATTGGTGCATTTGAGKTGATG-3′and probe Fam - TGAYCCAAAGCCTCTACTCAG TGC GAA AGC – Blackhole Quencher 1; seasonal influenza virus H3 gene forward primer: 5′-AAGCATTCCYAATGACAAACC-3′; seasonal influenza virus H3 gene reverse primer: 5′- ATTGCRCCRAATATGCCTCTAGT-3′ and probe Fam - CAGGATCACATATGGGSCCTGTCCCAG -Blackhole Quencher 1.

The determination of HA polymorphisms at residue 222 were performed by nested PCR. We first amplified a 568 bp fragment with outer forward primer: 5′-CAATGGAACGTGTTACCCAGGAG-3′and outer reverse primer: 5′- GCAATCGTGGACTGGTGTATCTGA-3′. We then amplified and sequenced an inner 380-base pair fragment spanning the position 222 of the pandemic H1 gene, as described previously [10]. The inner forward primer sequence was 5′-GTTCATGGCCCAATCATGAC-3′ and the inner reverse primer was 5′-GATTTCCAGTTGCTTCGAATG-3′.

### Statistical analysis

For statistical analysis, Fisher's exact test was used for comparing categorical variables and Mann-Whitney *U* test was used for continuous variables. p<0.05 was considered to represent statistical significance. McNemar's test with continuity correction was used to compare the frequency of D222G and other quasispecies in the NPA and blood samples. R version 2.11.0 (http://www.r-project.org/) was used for statistical analysis.

## Results

### Patients and clinical data

Data collected from a total of 139 patients were available for analysis. Among them, 85 patients were classified as severe cases according to the above definition and the remaining 54 patients were classified as mild cases. RT-PCR for seasonal influenza viruses was negative in all the patients. The median duration of symptoms before hospital admission was 3 days (range 1–18 days). Two patients developed symptoms during hospitalization. As noted in previous studies, patients with severe disease were found to be significantly older and more likely to have an underlying co-morbidity (data not shown). When patients were grouped according to the presence of viral RNA in different specimen sites, 119 patients were found to have positive viral RNA detection only in respiratory samples (NPA or ETA) while 20 patients have positive viral RNA detection in other sample types, including 14 patients with pandemic influenza A(H1N1) 2009 viremia whose blood samples were collected between 1 and 13 days after the onset of symptoms (Table 1). As expected, viremia was strongly associated with a severe clinical presentation (p = 0.0025; odds ratio = positive infinity). For the 6 survived cases and 3 deaths for whom the duration of viremia after hospital admission can be determined, the median duration was 3 and 2 days, respectively, but did not reach statistical significance (p = 0.179). As for the viral load in the blood, the mean viral load of blood samples with D222G/N quasispecies was 8310 copies per ml (range <900 to 29400 copies per ml), whereas the viral load for the sample without D222G/N quasispecies was 3950 copies per ml. Interestingly, there was no significant difference between the median ages of patients in the two groups despite the higher proportion of severely ill patients in the group with viremia. Patients with viral RNA detected only in respiratory specimens have significantly higher leukocyte counts (including differential counts) and platelet counts, but lower serum alanine transaminase levels. Though 24 patients had bacterial co-infections, none of them had viremia.

**Table 1:** Clinical, demographic and laboratory characteristics of hospitalized patients with pandemic influenza A/H1N1/2009 virus infection.

Characteristic	Patients with viral RNA detected in respiratory samples only (n = 119)	Patients with viral RNA detected in blood samples (n = 14)	p
Demographics and co-morbidities			
Age, in median years (range)	47 (18–85)	49 (19–59)	0.689
Sex			
Male	62 (52.1%)	7 (50.0%)	1.00
Female	57 (47.9%)	7 (50.0%)	
Underlying illness	85 (71.4%)	11 (78.6%)	0.757
Pregnancy (in women of childbearing age)	5 / 35 (13.3%)	0 / 4 (0.0%)	1.00
Laboratory findings on admission (median and range)			
Total leucocyte count, ×10^9^ cells/L	7.2 (0.8–32.05)*	4.4 (1.5–9)	0.0033
Neutrophil count, ×10^9^ cells/L	5.3 (0.33–30.45)†	3.1 (1.1–7.3)‡	0.0271
Lymphocyte count, ×10^9^ cells/L	0.8 (0.2–2.8)†	0.37 (0.1–1)‡	0.0004
Platelet count, ×10^9^ platelets/L	196 (13–400)*	152 (81–267)	0.0308
Alanine transaminase level, IU/L	20 (<6–261)§	58 (13–343)	0.0001
Clinical severity			
Severe	67 (56.3%)	14 (100%)	0.0025
Mild	52 (43.7%)	0 (0.0%)	
Clinical outcome (all cases)			
Survived	103 (86.6%)	8 (57.1%)	0.013
Died	16 (13.4%)	6 (42.9%)	
Clinical outcome (severe cases only)			
Survived	50 (75.8%)	8 (57.1%)	0.192
Died	16 (24.2%)	6 (42.9%)	

Patients with viral RNA detected in stool/ rectal samples but not blood (n = 6) were excluded from the comparison.

*(n = 117);

† (n = 113);

‡ (n = 11);

§ (n = 115).

### Molecular characterization of viral RNA outside the respiratory tract

Among the 20 patients with positive viral RNA detection in non-respiratory samples, 13 had viral RNA detected in blood while 6 patients had viral RNA detected in stool or rectal swabs. Only one patient was found to have viral RNA detected in both her blood and stool samples. We attempted to determine the viral HA gene sequence in all positive samples, though in some cases the RT-PCR for HA gene was negative despite a positive M gene RT-PCR result. In all cases, the only non-synonymous mutations detected at the partial HA gene sequence corresponded to polymorphism at amino acid residue 222 (Table 2).

**Table 2:** Viral hemagglutinin (HA) polymorphisms in specimens of patients with positive detection of pandemic influenza A/H1N1/2009 viral RNA outside the respiratory system.

Case no.	Sex/age	Clinical severity^a^	Clinical outcome	Viral load in blood sample (copies per ml)^b^	Polymorphisms at residue 222 of viral HA protein in various samples^c^			
					NPA	ETA	Blood	Stool/ rectal swab
1	F/45	+	Recovered	-	D	*(NA)*	–	N
2	M/62	+	Died	-	D	*(NA)*	–	D
3	M/59	+	Died	-	D	*(NA)*	–	D
4	F/50	+	Recovered	3.95×10^3^	D	*(NA)*	D	D
5	F/55	+	Recovered	-	D	*(NA)*	–	D
6	M/73	−	Recovered	-	D	*(Specimen not taken)*	–	D
7	M/51	+	Recovered	<900	D/G	D/G/N	D/G	–
8	M/51	+	Recovered	1.71×10^3^	D	D	*(NA)*	–
9	M/55	+	Died	6.71×10^3^	D	D	D/G	–
10	F/56	+	Died	6.40×10^3^	D	D	*(NA)*	–
11	M/41	+	Died	1.13×10^4^	D	D/G	D/G	–
12	F/34	+	Recovered	8.44×10^3^	D/G	D/G	G	–
13	F/56	+	Recovered	3.08×10^3^	D	D/G	N	–
14	M/19	+	Died	negative	D	D	*(NA)*	–
15	M/29	−	Recovered	-	D	*(Specimen not taken)*	–	D
16	F/43	+	Died	<900	D/G	G	G	*(Specimen not taken)*
17	M/47	+	Recovered	2.94×10^4^	D	D	N	*(Specimen not taken)*
18	F/59	+	Died	8.18×10^3^	D	D/G	G	*(Specimen not taken)*
19	M/43	+	Died	6.78×10^3^	D	D/G	D/G	*(Specimen not taken)*
20	F/43	+	Died	3.17×10^4^	D/G	D/G	*(NA)*	*(Specimen not taken)*
Total					D 20/20 (100%); G 4/20 (20%)	D 12/13 (92.3%); G 7/13 (53.8%); N 1/13 (7.7%)	D 5/10 (50%); G 7/10 (70%); N 2/10 (20%)	D 6/6 (100%)

a + = Severe cases; − = mild cases;

b Viral load is based on the quantitative PCR of the M gene. If multiple blood samples were tested, the highest viral load is shown;

c D = Aspartic acid; G = Glycine; N = Asparagine; – = matrix and hemagglutinin gene PCR negative; *(NA)* = HA sequencing unsuccessful.

**Figure 1:**
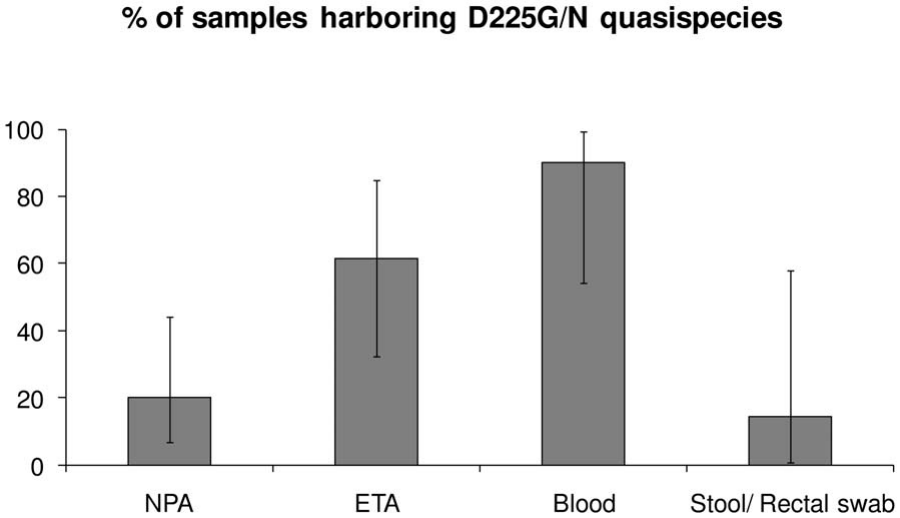
Proportion of samples with positive detection of D222G/N quasispecies in patients with positive viral RNA detection outside the respiratory system.

D222G and other quasispecies were found more frequently in blood samples than NPA samples of the same patients (McNemar's test with continuity correction, p = 0.041). This phenomenon was not seen in the positive stool/ rectal samples which recovered predominantly D222 quasispecies. D222N quasispecies was also found in 2 blood samples and 1 stool sample without co-detection of other quasispecies. Amongst patients with positive viral RNA detection outside the respiratory system, D222G/N quasispecies were observed in 20% of NPA samples, 61.5% of ETA samples, 90% of blood samples and 14.3% of stool/ rectal swab samples (Figure 1). Patient 4, for whom only D222 quasispecies were detected in blood, has a history of end stage renal disease.

## Discussion

Results of the present study highlighted the genetic heterogeneity of the pandemic influenza A(H1N1) 2009 virus quasispecies between the respiratory tract and blood in the same patient. Additionally, we found that the proportion of D222G/N quasispecies in blood is greater than the corresponding proportion in all other sampling sites. This suggests the possibility of differential anatomical site adaptation by the various pandemic influenza A(H1N1) 2009 virus quasispecies through polymorphisms at amino acid position 222. The only patient with only D222 detected in blood has end stage renal disease, which is associated with impaired leukocyte function [23]. To our knowledge, the D222G/N substitution is the first viral factor to be associated with influenza viremia, and it remains to be seen if the same substitution may confer the same phenotype to influenza strains other than the pandemic influenza A/H1N1/2009 strains.

The amino acid residues at positions 138, 190, 194, 225, 226, and 228 of HA (H3 numbering) are important for influenza A viruses to initiate infection via receptor binding [24]. Glycan microarray analysis of the pandemic influenza A(H1N1) 1918 virus found that receptor preference was determined by residues 190 and 225, with substitutions of D190E or D225G (H3 numbering) resulting in binding specificity to the avian-type receptor [25]. It is not entirely clear how the D222G/N or other mutations may contribute to the receptor specificity of the pandemic virus or its dissemination in the body. By correlating the RT-PCR results and polymorphisms observed at different sampling sites, we speculate that the virus, which normally replicates in the upper respiratory tract, adapts and replicates in the lungs through the D222G mutation and then disseminates into the bloodstream. The detected viral RNA in blood could either reflect extensive pulmonary damage with phagocytic uptake of virus-infected cells or true infection of monocyte-derived dendritic cells and macrophages [26]. On the contrary, viruses in the stool may originate from swallowed respiratory secretions, although viral replication in the epithelial tissue along the gastrointestinal tract cannot be ruled out entirely.

We have shown a higher leucocyte and platelet counts but lower serum alanine transaminase levels in patients with viral RNA detected only in respiratory specimens. The clinical significance and mechanisms underlying these differences are unknown, though we speculate that the observed differences may be a result of an increased cytokine response in patients with viral dissemination, or may simply reflect the severity of clinical disease at the time of dissemination [27].

The above results suggested that pulmonary damage remains a significant cause of mortality by the pandemic influenza A(H1N1) 2009 virus, unlike the avian H5N1 influenza virus which is typically associated with more systemic involvement. Given that avian H5N1 influenza virus has been demonstrated to replicate in anatomical sites outside the respiratory and gastrointestinal tract such as blood, cerebrospinal fluid and foetus, the difference in the impact of viral dissemination of the two viruses may have resulted from both difference in viral virulence and host response in terms of cytokines and chemokine levels. For clinical management purposes, presence of pandemic influenza A(H1N1) 2009 viremia is a useful indicator of disease severity but its prognostic value requires further validation. The defined D222G mutation in its HA protein is associated with bloodstream dissemination, extending our previous results which showed that the D222G mutation may have functional significance in the adaptation of the virus to the lungs. Although the occurrence of bloodstream dissemination does not always lead to a significantly worsened outcome, this could be related to the small sample size, especially in the viraemia cohort. We speculated that a bigger study will show a significant difference. Understanding the mechanism behind the responsible viral adaptation is important in the development of specific therapeutic strategies to prevent progression from a mild upper respiratory tract infection to severe pulmonary disease.

## References

[1] Chowell G, Bertozzi SM, Colchero MA, Lopez-Gatell H, Alpuche-Aranda C (2009). Severe respiratory disease concurrent with the circulation of H1N1 influenza.. N Engl J Med.

[2] Jamieson DJ, Honein MA, Rasmussen SA, Williams JL, Swerdlow DL (2009). H1N1 2009 influenza virus infection during pregnancy in the USA.. Lancet.

[3] Hung IF, To KK, Lee CK, Lin CK, Chan JF (2010). Effect of clinical and virological parameters on the level of neutralizing antibody against pandemic influenza A virus H1N1 2009.. Clin Infect Dis.

[4] To KK, Hung IF, Li IW, Lee KL, Koo CK (2010). Delayed clearance of viral load and marked cytokine activation in severe cases of pandemic H1N1 2009 influenza virus infection.. Clin Infect Dis.

[5] Gordon CL, Johnson PD, Permezel M, Holmes NE, Gutteridge G (2010). Association between severe pandemic 2009 influenza A (H1N1) virus infection and immunoglobulin G(2) subclass deficiency.. Clin Infect Dis.

[6] Mak GC, Au KW, Tai LS, Chuang KC, Cheng KC (2010). Association of D222G substitution in haemagglutinin of 2009 pandemic influenza A (H1N1) with severe disease.. Euro Surveill.

[7] World Health Organization (2010). Preliminary review of D222G amino acid substitution in the haemagglutinin of pandemic influenza A (H1N1) 2009 viruses.. Wkly Epidemiol Rec.

[8] Miller RR, MacLean AR, Gunson RN, Carman WF (2010). Occurrence of haemagglutinin mutation D222G in pandemic influenza A(H1N1) infected patients in the West of Scotland, United Kingdom, 2009–10.. Euro Surveill.

[9] Kilander A, Rykkvin R, Dudman SG, Hungnes O (2010). Observed association between the HA1 mutation D222G in the 2009 pandemic influenza A(H1N1) virus and severe clinical outcome, Norway 2009–2010.. Euro Surveill.

[10] Chen H, Wen X, To KK, Wang P, Tse H (2010). Quasispecies of the D225G substitution in the hemagglutinin of pandemic influenza A(H1N1) 2009 virus from patients with severe disease in Hong Kong, China.. J Infect Dis.

[11] Oughton M, Dascal A, Laporta D, Charest H, Afilalo M (2011). Evidence of viremia in 2 cases of severe pandemic influenza A H1N1/09.. Diagn Microbiol Infect Dis.

[12] Likos AM, Kelvin DJ, Cameron CM, Rowe T, Kuehnert MJ (2007). Influenza viremia and the potential for blood-borne transmission.. Transfusion.

[13] Kuiken T, Taubenberger JK (2008). Pathology of human influenza revisited.. Vaccine.

[14] Tsuruoka H, Xu H, Kuroda K, Hosaka Y (1997). [Viremia in influenza: detection by polymerase chain reaction].. Nippon Rinsho.

[15] Naficy K (1963). Human Influenza Infection with Proved Viremia. Report of a Case.. N Engl J Med.

[16] Poliakova TG, Ketiladze ES, Zhilina NN, Stakhanova VM (1970). [Viremia in influenza A2 (Hong Kong)].. Vopr Virusol.

[17] Stanley ED, Jackson GG (1966). Viremia in Asian influenza.. Trans Assoc Am Physicians.

[18] Minuse E, Willis PW, Davenport FM, Francis T (1962). An attempt to demonstrate viremia in cases of Asian influenza.. J Lab Clin Med.

[19] Herzberg K, Vanek E, Reuss K (1961). [Research on the problem of influenza viremia. II. Virus detection experiments on the blood of human influenza patients].. Z Hyg Infektionskr.

[20] To KK, Wong SS, Li IW, Hung IF, Tse H (2010). Concurrent comparison of epidemiology, clinical presentation and outcome between adult patients suffering from the pandemic influenza A (H1N1) 2009 virus and the seasonal influenza A virus infection.. Postgrad Med J.

[21] Chan KH, Peiris JS, Lim W, Nicholls JM, Chiu SS (2008). Comparison of nasopharyngeal flocked swabs and aspirates for rapid diagnosis of respiratory viruses in children.. J Clin Virol.

[22] Centers for Disease Control and Prevention (CDC) (2007). CDC Realtime RT-PCR (rRTPCR) protocol for detection and characterization of influenza (version 2007)..

[23] Vanholder R, Ringoir S (1993). Infectious morbidity and defects of phagocytic function in end-stage renal disease: a review.. J Am Soc Nephrol.

[24] Skehel JJ, Wiley DC (2000). Receptor binding and membrane fusion in virus entry: the influenza hemagglutinin.. Annu Rev Biochem.

[25] Stevens J, Blixt O, Glaser L, Taubenberger JK, Palese P (2006). Glycan microarray analysis of the hemagglutinins from modern and pandemic influenza viruses reveals different receptor specificities.. J Mol Biol.

[26] Osterlund P, Pirhonen J, Ikonen N, Ronkko E, Strengell M (2010). Pandemic H1N1 2009 influenza A virus induces weak cytokine responses in human macrophages and dendritic cells and is highly sensitive to the antiviral actions of interferons.. J Virol.

[27] Rouphael NG, Talati NJ, Vaughan C, Cunningham K, Moreira R (2007). Infections associated with haemophagocytic syndrome.. Lancet Infect Dis.

